# Office Workers’ Views About the Uses, Concerns, and Acceptance of Hand Hygiene Data Collected From Smart Sanitizers: Exploratory Qualitative Interview Study

**DOI:** 10.2196/47308

**Published:** 2024-01-11

**Authors:** Sophie Rutter, Sally Sanger, Andrew D Madden, Sukaina Ehdeed, Catherine Stones

**Affiliations:** 1 Information School University of Sheffield Sheffield United Kingdom; 2 School of Design University of Leeds Leeds United Kingdom

**Keywords:** hand hygiene, smart sanitizers, Internet of Things, IoT, offices, workplaces, smart systems

## Abstract

**Background:**

COVID-19 and the prospect of future pandemics have emphasized the need to reduce disease transmission in workplaces. Despite the well-established link between good hand hygiene (HH) and employee health, HH in nonclinical workplaces has received little attention. Smart sanitizers have been deployed in clinical settings to motivate and enforce HH. This study is part of a large project that explores the potential of smart sanitizers in office settings.

**Objective:**

Our previous study found that for office workers to accept the deployment of smart sanitizers, they would need to find the data generated as useful and actionable. The objectives of this study were to identify (1) the potential uses and actions that could be taken from HH data collected by smart sanitizers (2) the concerns of office workers for the identified uses and actions and (3) the circumstances in which office workers accept HH monitoring.

**Methods:**

An interview study was conducted with 18 office workers from various professions. Interview questions were developed using a framework from personal informatics. Transcripts were analyzed thematically.

**Results:**

A wide range of uses of smart sanitizer data was identified including managing hygiene resources and workflows, finding operating sanitizers, communicating the (high) standard of organizational hygiene, promoting and enforcing organizational hygiene policies, improving workers’ own hygiene practices, executing more effective interventions, and identifying the causes of outbreaks. However, hygiene is mostly considered as a private matter, and it is also possible that no action would be taken. Office workers were also concerned about bullying, coercion, and use of hygiene data for unintended purposes. They were also worried that the data could be inaccurate or incomplete, leading to misrepresentation of hygiene practices. Office workers suggested that they would be more likely to accept monitoring in situations where hygiene is considered important, when there are clear benefits to data collection, if their privacy is respected, if they have some control over how their data are collected, and if the ways in which the data will be used are clearly communicated.

**Conclusions:**

Smart sanitizers could have a valuable role in improving hygiene practices in offices and reducing disease transmission. Many actionable uses for data collected from smart systems were identified. However, office workers consider HH as a personal matter, and acceptance of smart systems is likely to be dynamic and will depend on the broad situation. Except when there are disease outbreaks, smart systems may need to be restricted to uses that do not require the sharing of personal data. Should organizations wish to implement smart sanitizers in offices, it would be advisable to consult widely with staff and develop systems that are customizable and personalizable.

## Introduction

### Hand Hygiene in Workplaces

Recent pandemics (such as severe acute respiratory syndrome 1, H1N1 influenza, Middle East Respiratory Syndrome, and COVID-19) have demonstrated that public health threats are synonymous with occupational health threats [1]. Good hygiene protects people from acquiring and spreading gastrointestinal infections and respiratory infections [2]. When employees work in close proximity with others (colleagues, clients, and customers), share spaces (such as offices, kitchens, and break rooms) and share resources (such as computers, photocopiers, water coolers, and sinks), infectious diseases can be easily spread [3]. Hygiene practices such as using sanitizer, washing hands, and disinfecting surfaces have proved to be effective in reducing pathogen spread [4,5] and reducing illness in workplaces [3] and may be adopted more readily than other public health measures such as mask wearing [6].

Several studies have found that good hand hygiene (HH) reduces both absenteeism and presenteeism (attending work when ill), increases productivity [3,7], reduces the pressure on health services, and helps to tackle antimicrobial resistance through the reduced use of antibiotics [8]. How much time is lost to work owing to poor hygiene is difficult to quantify [9] as hygiene-related absences may be brief organizations tend not to report them to authorities, and it is often not possible to directly connect an acquired illness with poor hygiene practice (eg, without specific tests, what led to an employee’s stomach upset is speculative). Nonetheless, many of the studies cited previously indicate that much time is lost to work because of poor hygiene practices. Furthermore, before COVID-19, for many workers, attending work was rarely considered a risk to health, but now, employees may be highly anxious about infections in the workplace [10].

The COVID-19 pandemic led to a proliferation of guidance about reducing infection transmission in the workplace [7,11,12]. People were, and still are in some settings, encouraged to work from home where possible, keep socially distant, wear masks, wash and sanitize hands, and get vaccinated. Workplaces can also use 3 strategies to control infections [13]. First, they can try to prevent infections from entering the workplace through health screenings and by reducing or eliminating contact between workers (eg, working-from-home policies). Second, workplaces can help stop the transmission of infection through ventilation strategies and by erecting barriers and screens to prevent movement of aerosols. Finally, workplaces can help protect the worker from acquiring infections with personal protective equipment. However, despite many of these measures being introduced into workplaces, infectious disease transmission remains as a challenge [1]. This matters because it is vital that workplaces are in a position to adopt infection prevention and control strategies as and when required for current and future infectious diseases [1].

### Smart Sanitizers

Smart sanitizers are already on the market and deployed in clinical settings. In this study, we consider the potential of smart HH systems in offices. Also known as automated hand hygiene monitoring systems (AHHMS) and electronic monitoring systems, smart sanitizers are Internet of Things devices. The device (the “thing”) stores and dispenses soap when activated by a sensor. Sensors within the device collect information about soap consumption (activation of the soap dispenser and fill level of the dispenser). When networked with other sensor data, such as movement of people (eg, entry to a room or building) and person tags (such as staff ID cards), the smart system can monitor a person’s HH based on where they are and what they are doing. The individual or aggregated data can then be shared on personal devices and apps, with sanitizer users and anyone else on the network. The system can also send messages and reminders and give feedback to registered users. The basic functionality has been established for >10 years and continues to develop and evolve. Recently, there has been a move to develop smart systems that can measure hand washing quality (correct technique and adequate time) [14].

Smart sanitizers have been adopted in clinical settings where HH is operationally crucial to help stop the spread of health care–associated infections, and there is a requirement to audit health care workers’ HH when caring for patients [15,16]. In clinical settings, smart sanitizers are generally considered to be effective in increasing HH, at least in the short term [17]. As HH is an important part of health care workers’ professional practice, many health care workers welcome the use of technology to improve hygiene adherence [18,19]. However, there are concerns about the loss of privacy and the potential for coercion, with many health care workers expressing a preference for systems that do not collect any personal data [14,19,20]. Health care workers are also concerned that the data collected may not accurately represent hygiene practices if the technology is prone to error, deliberately manipulated, or the context of HH (or rather, lack of HH) is not taken into account [14,18-20]. Furthermore, there are concerns about infrastructure costs and the potential for side effects of using systems that use radio frequency interference and UV light [14].

There has been little deployment of smart sanitizers outside clinical settings. Whether office workers would be as open as health care workers to adopting this technology needs further investigation, particularly because acceptance of smart sanitizers is dependent on organizational culture and how monitoring is implemented [17,18]. Moreover, as the professional concerns differ, how the technology is deployed and used in office settings may be different.

In 2021, Zivich et al [21] conducted a feasibility study for collecting HH data in offices and data about person-to-person contacts. Sensors were installed in soap and alcohol sanitizers in 2 US offices, and those participating (n=43) also carried sensors. From the data collected, first, the study authors found that office workers likely overestimate the frequency of their HH practices and, second, those with supervisory roles had fewer in-person interactions than those without supervisory responsibility. The authors also found that study participants were willing to carry sensors and have their interactions tracked. However, participation in the study was not obligatory and those participating were appropriately incentivized with a US $25 gift card. It is therefore not clear whether these office workers would be happy with such tracking as part of their usual working practices, and in fact, some study participants suggested that they would need an increase in compensation to participate in a long study. Together, these findings suggest that smart sanitizers could be useful in (1) helping office workers identify their HH practices and (2) understanding and managing disease transmission in offices. However, it is not clear whether office workers would be willing in everyday life to have their hygiene data and contacts with other people collected.

Further to this, we investigated the attitudes toward the use of smart sanitizers in the workplace using a survey of workers in nonclinical settings (n=314), followed up with a qualitative questionnaire (n=12) and interview (n=3) [22]. Survey participants were generally in agreement that at work, high standards of HH is important and that smart sanitizers could usefully inform maintenance staff when to refill. However, there was little consensus with regards to the acceptance of collecting data that would give office workers an overview of their own HH practice, allow them to compare their own practices with those of others, provide them with personal messages, and give managers an anonymized view of HH practices. What was clear from the written responses and interviews is that participants thought it important that the data should only be collected if they can be acted upon, that is, the data should not just be collected because the technology allows it. This means that, before introducing smart sanitizers to the workplace, it is necessary to identify what actions could be usefully informed by the data. This led to our first research question (RQ), for which we adopted an exploratory approach to identify all the potential actions: RQ1—What actions could be taken from HH data collected by smart sanitizers?

However, whether these actions would be accepted by office workers requires further investigation, because survey participants were also concerned that HH data could be misused and misinterpreted. In particular, participants were concerned that collecting HH data could be an invasion of privacy, and the data collected may not be accurate. What HH data are needed will depend on how the data are to be used; therefore, it would be helpful to know the concerns associated with possible actions. This led to our second RQ: RQ2—For the actions identified, what, if any, are the data collection concerns of office workers?

Finally, the survey was conducted during the pandemic (July 2021 to August 2021), at a time when participants may have considered HH as particularly important. Concern for their health could have influenced the extent to which participants were willing to accept monitoring. When else, if ever, office workers would be more likely to accept smart sanitizers is not known. This led to our final RQ: RQ3—Under what circumstances would office workers accept HH monitoring?

## Methods

### Overview

This study was conducted as part of a large project to develop a smart hand sanitizer for the office environment. The project is a collaboration between the University of Sheffield (Information School) and the University of Leeds (School of Design), together with Savortex (a manufacturer of HH technology). The study reported in this paper, including data collection and analysis, was conducted solely by the universities.

### Recruitment

This was a qualitative study to identify the potential uses of smart sanitizers from the perspective of those who work in offices all or most of the time. Interviews were conducted either using video link or via telephone, and they occurred between January 2022 and March 2022. The questions were pilot-tested with 2 participants known to the project team. Participants from a previous survey of attitudes toward the use of smart sanitizers in the workplace [22], who had expressed interest in further participation, were invited to participate in this study: 11 participants consented. To elicit a range of views, additional 7 participants were recruited using the research team’s networks. Although half of the participants (9/18, 50%) were from the education sector, sector did not account for differences in responses in the previous survey [22]. There were 18 participants in total, 3 (17%) of whom had some responsibility for hygiene within their organization (Table 1).

**Table 1:** Distribution of study participants based on role, sector, and responsibility for hygiene.

Participant number	Role	Sector	Responsibility for hygiene
P1	Health care professional (office based)	Private health services	No
P2	Educator	Education	No
P3	Administrator	Local government	No
P4	Administrator	Health services	No
P5	Researcher	Education	No
P6	Social worker	Local government	No
P7	Educator	Education	No
P8	Disability liaison officer and educator	Education	No
P9	Conveyancer	Legal	No
P10	Director	Research and design	No
P11	Facilities manager	Education	Yes
P12	Hearing impairment teacher	Education	No
P13	Not known	Media and culture	No
P14	Deputy facilities manager	Education	Yes
P15	Facilities manager	Soft service industry	Yes
P16	Educator	Education	No
P17	Finance officer	Local government	No
P18	Educator	Education	No

### Data Collection

To prepare for data collection, we turned to the field of personal informatics. Personal informatics systems “help people collect personally relevant information for the purpose of self-reflection and gaining self-knowledge” [23]. As such, smart HH systems can also be considered as personal informatics systems because individuals can use them to collect and track data about their HH practices. A semistructured interview guide was developed based on the stage-based model by Li et al [23]. This model is widely used in the design of personal informatics systems and holistically describes, from a user perspective, the stages of collecting and using personal data. A set of main questions (Table 2) relating to the 5 stages (preparation, collection, integration, reflection, and action) was prepared, together with several possible prompts. To allow for the identification of all the potential uses of HH data, we did not restrict the discussion to smart sanitizers that are currently on the market; at the beginning of the interview, participants were told that “By hand hygiene we mean using any kind of hand cleaning facility. This includes hand washing, using a wall sanitiser or using your own sanitiser from a bottle or a wipe.”

**Table 2:** Study interview guide based on the stage-based model of personal informatics by Li et al [23].

Stage-based model of personal informatics	Main question
Preparation	How could hand hygiene data be used and what data should be collected?
Collection	How should hand hygiene data be collected?
Integration	How should the collected data be prepared and processed?
Reflection	Who should see the data and how should this be presented?
Action	What might you do as a result?

### Data Analysis

Data were analyzed inductively using a “codebook” approach to thematic analysis [24], whereby a structured coding framework is used to analyze the data. Preliminary open coding was performed by the second author. At a follow-up meeting, developing codes were discussed with the first, third, and fourth authors and an initial codebook was compiled. The second author completed the coding of the remaining transcripts. The first author mapped the open codes to the RQs. This was then further reviewed by the second author.

### Ethical Considerations

This study received ethics approval (038337) from the University of Sheffield Research Ethics Committee on February 16, 2021. All study participants received an information sheet about the project, and they were given opportunities to ask questions and advised that they could withdraw with no negative consequences. All participants gave their informed consent. For confidentiality, their data are anonymized.

## Results

### RQ1: What Actions Could Be Taken From HH Data?

#### Overview

An exploratory approach was adopted for the first RQ, and all potential actions were identified. It was thought that HH data could be acted upon to (1) manage hygiene resources and workflows, (2) find operating sanitizers, (3) communicate the (high) standard of organizational hygiene, (4) improve own practice, (5) promote an organization’s hygiene policy, (6) enforce organizational policy, (7) target the training according to needs, (8) execute more effective interventions, and (9) identify the causes of outbreaks, and whether (10) any action would be taken was also considered.

#### Manage Hygiene Resources and Workflows

Facilities managers could find HH data useful when planning and maintaining hygiene facilities, including the purchase and decommissioning of sanitizers, purchase of soap and gel, optimal placing of sanitizers, and identification of when maintenance is required. Maintenance data could also make work processes and workflows more efficient:

We then don’t have to send a cleaner every hour for no reason.

P15

#### Find Operating HH Facilities

If stock fill level data were shared with everyone, building users could act upon the data to find operating HH facilities:

It’s a bit like “FindmyPC” isn’t it?... If there’s nothing [soap and gel] on [place] I can go to [place] and I can get the stuff there.

P11

#### Communicate the (High) Standard of Organizational Hygiene

Organizations could use HH data as tangible evidence to reassure employees and visitors that there is a high standard of HH in the building. This could be particularly useful for organizations that work with vulnerable people:

This last calendar month we had 95% usage of all of our machines [that would communicate] we’re looking after our staff and the compliance of that.

P15

#### Improve Own HH Practice

Through managing, tracking, and understanding their hygiene practices, including evaluating their HH technique, individuals could act upon the data to improve their HH practices:

Like, let’s say you have a ring, it’s not very clean around the ring, I would then know and I would spend more time obviously.

P13

If the system collected contextual data (including what the person is doing at the time, how they are feeling, and the current risk of catching an infection), the system could usefully identify trends, give insights, and make recommendations that would enable individuals to further act upon their HH data:

Recommendations on my hand washing behaviour, like, you know: “In general, you do not seem to wash your hands very well on Tuesdays or on Wednesdays.” That might help me understand why that’s the case. Another thing that might be useful...then it might be nice to know if I’m washing hands when I’m meeting people more or if I have more meetings. So if it’s connected with the calendar then it might be able to give some more insight into why I think I’m not washing my hands more, when I’m washing them and where I’m going [next] so that might be good.

P2

Participants thought that having access to other people’s HH data could enable people to benchmark and contextualize their own results:

If I could compare my handwashing with somebody else’s, and if mine looked that I was hand-washing too often, then I’d have to look at if we’re all doing the same number of visits in a day, am I sanitising my hands too much, but then if I’m not getting infections at the same rate as other people then maybe I’m not hand sanitising my hands too much.

P12

#### Promote an Organizational Hygiene Policy

Organizations can use smart systems to communicate their HH policy. Smart systems could help promote policies by sending reminders and keeping employees motivated through comparisons, competitions, and other incentives:

I suppose they should have some sort of benchmark, you know, like “The rest of the organisation are all doing it really frequently and doing it for the right duration, but your team aren’t” so they have to have some sort of like benchmark as to where they fall on a scale, as it were.

P4

It was thought that smart systems could be useful when new routines are introduced:

If there were changes in expectations, such as more restrictions were put in place, if there was another outbreak.

P6

This would also apply when new staff join the organization.

#### Enforce Organizational HH Policy

Organizations can use smart systems to identify compliance and changes in compliance. If a lack of compliance has been identified, organizations could target particular events (such as after using the toilet), individuals, teams, and departments to set hygiene goals that align with their policy:

Showing trends, showing ups and downs, especially the downs, might highlight points to people to make them realise...you can use that to some effect then, can’t you, if you have a particular outbreak in a particular team or whatever. It might prompt people to take a bit more action to it maybe.

P9

The data could also be synced with door entry systems to prevent people from entering spaces (such as food preparation areas), but none of the participants (0/18, 0%) thought this was a good idea.

#### Target HH Training According to Needs

An analysis of HH data could also help to identify who needs training and what their training needs are:

If they’re looking at training needs and compliance and safety and all those sorts of things, could use those to identify if there are any gaps.

P4

#### Execute More Effective Interventions

Organizations and researchers could evaluate the effectiveness of interventions in real time and adapt them according to the results:

It doesn’t have to be Coronavirus, it could be the flu or something, it would be useful to see that, and to see how people responded to prompts and reminders.

P6

#### Identify the Causes of Outbreaks

Participants also discussed the possibility that if HH data were combined with other health data, it could enable researchers to gain a better understanding of the impact of HH on health and the cause of infectious outbreaks:

Reporting that there’ve been a lot of stomach upsets, and that was linked in with the data on hand washing, which was very low, then you could put two and two together, and that could be useful.

P5

#### No Action

Whether any action would be taken was also discussed. HH was often thought to be a personal matter and the responsibility of the individual. Several participants stated that they would not say anything or take any action if they knew their colleagues had inadequate HH practices:

I do think it’s pretty disgusting if people don’t wash their hands, [pause] but it’s not for me to tell them to...I’ve just got to be responsible for myself.

P17

### RQ2: For the Actions Identified, What, if Any, Are the Concerns of Office Workers?

Office workers are concerned that (1) intentions and messages could be misinterpreted and that data could be used for (2) bullying and coercion, (3) unintended purposes, (4) inaccurate representation of HH practices, and (5) incomplete representation of HH practices. Next, we have discussed which actions raise the concerns.

#### Intentions and Messages Are Misinterpreted

It was thought that messages generated by a smart sanitizer may not be received as intended. Using HH data to reassure building users about the status of HH in organizations could instead make them feel anxious:

Then again it could let people, like I say, who are socially anxious think “Oh my God, no-one’s cleaning their hands, it’s a really dirty place.” You will get people that will freak out about that.

P3

Although organizations may install smart HH systems to reassure office workers, office workers may feel that monitoring could imply that a person is not able to manage on their own. The installation of devices that monitor HH could be construed as a message conveying lack of trust:

It felt like if we monitored something like that, then it would damage trust, it would make people less independent and capable of taking care of their health because it would set an expectation that someone else is going to monitor it.

P10

#### Bullying and Coercion

Many interviewees felt that the data would be of particular interest to managers, but using the data to promote and enforce HH policy could lead to bullying, be divisive, and encourage rivalries:

I’d be concerned in some bits of the organisation that I worked in, that some managers would use it punitively to, not necessarily call out people publicly, but use it to...bully people or shame them or whatever.

P4

Benchmarking one’s own HH against others was thought to be helpful in improving one’s own HH practice, but it was also thought that office workers may feel harshly and unfairly judged:

If my whole team does it, then if I don’t do it then I’m gonna surely [be] judged for, like, not cleaning my hands even though it [my reason for refusing] has nothing to do with that.

P13

#### Used for Unintended Purposes

Participants thought that HH data could be (deliberately or inadvertently) used for purposes that do not benefit the organization or their employees. Moreover, HH data may reveal other personal information that would not be appropriate for organizations to know:

You might feel forced to say, “Oh, actually, I’ve got a bit of morning sickness. I think I might be pregnant,” and then you might have a miscarriage or something like that, so then it could – that might all – oh, dear, yeah. Or you might – say it could be an emotional reason why you’re going to the toilet. You might be going because you’re very upset about something. But I think, yeah, it could reveal all sorts of things about human behaviour, and actually, in an unintended way, reveal things about that person that are very private.

P5

There was some concern that manufacturers of sanitizers and cleaning products would use the data to increase sales:

If the outcome is, how can we sell more hand sanitizer, what if we connected our hand sanitizer product to the internet...I don’t think that’s a good outcome, and I don’t think it comes from a good place.

P10

#### Inaccurate Representation of HH Practices

Whether the data collected would be an accurate representation of HH practices and, therefore, whether any conclusions can be drawn from the data collected was also a concern. Participants worried that the use of smart systems could be manipulated, and therefore, the resulting data would be inaccurate and would misrepresent organizational HH practices:

I know some people who are just going to go round and just put their hand under every time they walk past just so they’ve triggered it whether they’re washing their hands or not.

P12

#### Incomplete Representation of HH Practices

Participants expressed the concern that smart systems could not capture all the data necessary to represent all HH practices, and this also adds to concerns about whether any conclusions can be drawn from the data. First, smart systems alone cannot capture all HH events (such as an employee’s use of their own sanitizer and wipes, which may be a personal preference or a necessity, eg, if a person has allergies to a particular substance):

Maybe you think, “I don’t want to touch the wall hand sanitizer because everybody else has touched that, so I’m going to stick to my personal sanitizer,” in which case, that wouldn’t capture any of that, so you would need both, for a true figure.

P5

Second, employees may work from home or in other locations outside the aegis of the organization, where it would be difficult for smart systems to capture HH events. Furthermore, for improving their own HH practices, people would want data beyond the work context:

How long I’m spending washing my hands, gaps in between, but also if there’s any variation in days. So, I mean, Saturday and Sunday might not be different if I’m out and about, on a personal level, than the Thursday or Friday if I’m working. I would expect there to be, but if I was shopping, and I went in 20 shops on a Saturday, that might reflect that I was handwashing the same as I was in a working day.

P12

Finally, to fully interpret HH practices, it would also be necessary to collect data about what the employee was doing at the time and where. Otherwise, there is a danger that the system may incorrectly interpret HH practices as missing. Participants also questioned whether using HH data to draw comparisons between different departments would be meaningful, as different roles may have different HH requirements:

If I’ve just come from the toilet and I’ve washed my hands and walked past a hand sanitiser, if I got a pop up on my machine...that says “You’ve been past a hand sanitiser and you haven’t used it” I would expect to be able to interact with it and explain to it why I haven’t used it.

P4

If it went like comparing groups within an organisation, then how do you know you’re comparing like with like?

P5

### RQ3: Under What Circumstances Would Office Workers Accept HH Monitoring?

The concerns expressed previously suggest that there will likely be some resistance to many of the uses of HH data. However, office workers suggested that they are more likely to accept HH monitoring (1) if they or others could not be identified, (2) in situations where HH is considered important, (3) when events considered as private are not recorded, (4) when data collection can be customized, (5) when data are used for a beneficial purpose, and (6) when uses of the data are clearly communicated.

#### When Identity Is Protected

Participants expressed little or no concern about organizations accessing data from sensors in which no personal data are collected and were therefore generally accepting of the uses of HH data for resource planning purposes.

Most participants thought that data about individuals should not be shared with others. However, 11% (2/18) of the participants thought that attributed personal data should be seen by senior managers (P4 and P6) and another 11% (2/18) thought that attributed personal data could be seen by team managers also (P12 and P14):

So with the data and reports, the only things that I think that someone else should be seeing about me are aggregate. So nothing where people can be identified.

P2

Senior management team or board level or Health and Safety Executive should have all the information by teams or by individuals but the individual managers...I wouldn’t want my manager of my department to have individualised data that makes them be able to say “[name] is not washing her hands often enough.” I would prefer that it’s anonymised at that level.

P4

Although participants were generally uncomfortable with personal data being shared with others, they were mostly comfortable with personal data collection if they or their colleagues could not be identified in any reports:

I think aggregated reports should be available to everyone, as a comparison purpose. I think maybe a little bit more detailed aggregated reports, for example, with a maximum, minimum, with a band, with a percentile band, with the longer period of change can be available to health and safety officer, can be a department manager, or what they call the senior manager group, steering committee.

P8

#### Situations Where HH Is Considered Important

Monitoring was thought to be more acceptable when the importance of HH is clear. Therefore, monitoring was seen as more acceptable in certain settings, notably, health care and food preparation, and for certain teams or roles, for example, food technicians and carers:

If I worked in a food environment, it’d be very different.

P17

It was also thought more acceptable during infectious disease outbreaks:

Suppose there’s another virus outbreak and it’s demonstrated that hand washing is key to preventing its spread, and that you’re doing it for the public good...if it was, like, three years ago, I would’ve said this is ridiculous. Now, I think maybe, OK, in the right circumstances, I would go along with it, because the context seems to have changed.

P7

It was thought to be acceptable at places in buildings where HH is important such as food preparation areas and toilets:

I would like to know that the people preparing my food wash their hands, that would be a good thing to know, because it’s crucial for there. The rest of them, I don’t need to know that, I don’t think...Although, I would prefer it if people washed their hands before they left the toilet – if an alarm went off there.

P5

#### When Events Considered Private Are Not Recorded

Although it was thought helpful to capture HH data in locations where HH is important, data capture was felt to be more acceptable in some parts of the building than others. For example, monitoring HH on building entry was less controversial than monitoring outside a toilet:

Some people might think that it’s a bit of an invasion of privacy, being monitored in the toilet as well. Is there nowhere safe? Is there nowhere that I can just not be monitored?

P4

#### When Data Collection Can Be Customized

It was thought that office workers would be more likely to accept monitoring if they can customize the system and control what data are collected and how they are presented:

I think perhaps like with the alerts, perhaps [they] could have the option to turn that on [recommendations, encouragement, advice] if you so desired, but it shouldn’t be a requirement.

P5

The system should allow users to correct any errors in data collection and add explanations, so that managers do not unfairly target individuals:

So that you’ve got the chance to correct yourself if you need to, like, you see I would be going back to my computer and I would expect then to have a message on my computer that says “You’ve walked past a hand sanitiser and you didn’t use it. What was the reason?”

P4

#### When Data Are Used for a Beneficial Purpose

Participants felt that data need to be collected for a purpose. The purpose needs to be justified, and the data should be retained only for as long as necessary. Furthermore, systems should be used to support individuals rather than punish them:

If there was a real, proper reason that they were collecting it for, then they could collect it for the relevant time period. So if there was some sort of disease outbreak and it lasted six months, then collect it for six months...it has to be justifiable...it’s not right to just collect it and hold that data.

P5

It depends on what people perceive is the overall intention of whoever’s putting this policy in place. If it’s used--, if the perception is it’s used to beat people up about hygiene because it’s going to lead to a poor sick record or more transmissible covid than that is a different intention to “Well, I’m really bothered about how sore your fingers are becoming with all your hand washing.”

P12

#### When Uses of the Collected Data Are Clearly Communicated

Participants recommended that the uses of the data need to be transparent and clearly communicated including how the data will be used and reported, who has access to what data, where and for how long the data are stored, and whether it is possible to opt out of data collection:

Why they’re doing it, who’s going to hold the data, who’s going to see it, how’s it going to be reported, who will it be shared with, yeah, where will the data go, how long will they hold the data for, and can I opt out, how do I opt out.

P2

## Discussion

### Principal Findings

Our previous study found that office workers thought HH data should only be collected if they can be acted upon [22]. In this study, office workers were able to identify several actions that could usefully be informed by HH data (RQ1). These included using the data to manage hygiene resources and workflows, find operating sanitizers, communicate (high) organizational standards of hygiene, improve workers’ own practice, promote and enforce an organization’s hygiene policy, target the training according to needs, execute more effective interventions, and identify the causes of outbreaks. However, hygiene is mostly considered as a private matter, and it is possible that no action would be taken in practice. Furthermore, office workers expressed concerns (RQ2) that the data could be used to bully, to coerce and for unintended purposes. Moreover, the data could be misinterpreted, inaccurate, and an incomplete representation of hygiene practices. Office workers suggested that they would be more likely to accept monitoring for the identified uses (RQ3) when their privacy is respected, they have some control over how their data are collected, and how their data will be used is clearly communicated. Monitoring is also more likely to be accepted in situations where hygiene is considered important and there is a clear beneficial purpose for data collection.

### HH Is (Mostly) a Personal Matter for Office Workers

Although the findings of this study suggest that facility managers, health and safety officers, departmental managers, building occupants and visitors, hygiene resource suppliers, researchers, and those interested in public health would find HH data useful, HH was thought to be a personal matter [25]. Several office workers reported that they would not take any action if they found that their colleagues’ HH practices were inadequate. For all the uses of HH data, there was a strong preference for personal data to be anonymized or not collected at all. However, acceptance of HH monitoring is dynamic and dependent on the situation and the context within which the data are used. The findings of our study suggest that it is much more likely to be accepted during disease outbreaks, in certain locations (eg, entrance to buildings), and in sectors (eg, health and food) where HH is important to the ethos and culture of the organization (ie, will also influence office workers). Although none of the office workers (0/18, 0%) thought it acceptable to enforce HH by restricting access to areas (by syncing HH data with door entry system data), it is possible that under extreme circumstances and in certain locations, this could be acceptable.

### Need for a Shared Understanding of “Good” Office HH

For those concerned about catching infectious diseases in the workplace [10], HH data could be used to reassure visitors to a building about the high standards of hygiene within the building and to promote and enforce organizational HH policies. In health care settings, smart sanitizers are already used to audit compliance and enforce the health sector’s policy of sanitizing hands before, during, and after patient care. Overall, 3 factors are likely to make smart sanitizers more acceptable in health care settings. First, they are used to enforce an HH policy that is considered important in professional practice [18]. Second, smart sanitizers can collect data that measure the compliance with policies such as the 5 moments for hygiene [26] that can be measured using the sanitizer supplied by the organization. Third, at least at a basic level, adherence to this policy can be monitored using room and sanitizer sensors, without the need for personal data collection.

For smart systems to be adopted in offices, it would help if there were an agreed-upon understanding of what is good hygiene practice, for example, how often and where (eg, entrances to buildings and exits from toilets) hygiene should be performed. Smart systems could then be used to reassure and promote HH in offices, if the policy can be complied with using office resources and without the need to collect personal data.

### Office Workers Want Insights From All Their HH Practices

Health care workers may wish to track their HH practices around patients, as good HH is part of their professional identity [18], and having access to their personal HH data could help health care workers improve their HH practice [27,28]. Given that office workers are likely to be overestimating their HH practices [21], self-tracking could be beneficial. However, office workers in this study did not link self-tracking of HH with professional expectations and standards; rather, they were interested in gaining insights into their overall HH practices including in all locations (office, home, and when they are out of the office and across all facilities (sanitizer, soap, wipes, etc). No smart system (as yet) can automatically detect HH with such detail. This would only be possible if office workers were prepared to input data manually, and this would require considerable motivation.

### Office Workers Share Health Care Workers’ Concerns

Many of the concerns that office workers expressed are similar to those of health care workers. Similar to health care workers [19,20], most office workers are concerned that personal data could be misused to punish or bully employees, and use of HH data should be clearly and transparently communicated. It would be advisable to consult with office workers early in the system design process to engage them, explain what purposes the organization intends for the data, and identify what purposes they feel are acceptable and useful.

Another concern shared with health care workers is whether smart HH systems can accurately represent HH practices, because, first, systems can be gamed and deliberately misused, and second, systems may incorrectly interpret events as missed HH opportunities because they are not registering the wide context within which the event did or did not occur [18-20]. Given the shift to more hybrid and flexible working [29], it may also be necessary to analyze HH data alongside work patterns. More generally, studies of workplace tracking have found that systems that enable employees to customize and control what data are collected are more likely to be accepted [30].

Office workers were also concerned that the data could be used for purposes other than what it was intended for. This is understandable given that misuse of technologies is widely reported in the media; for example, AirTags designed to track property have been used to stalk individuals [31].

### Useful for Health Researchers

Using sensors to remotely collect HH data resolves some of the challenges for health researchers who need to evaluate hygiene interventions. Researchers may evaluate interventions by observing HH practices, but the presence of an observer may change the behavior of the person being observed, particularly because HH is a social norm. Instead, researchers may use proxy measures such as changes in soap consumption. However, manually collecting soap consumption data from organizations is time consuming, and sensors can help in saving time [32].

Although it is thought likely that poor hygiene could contribute to disease transmission in workplaces, little data are available to support (or oppose) this point [9]. Connecting smart HH system data with other health data (such as data relating to employee absence) could help researchers understand the relationship.

Data collected from digital technologies (such as mobile phones, social media networks, and search engines) have been used to communicate public health messages and monitor and control outbreaks [33]. Smart HH systems could usefully be added to the arsenal of digital data sources that have been used to support health authorities’ response to COVID-19 and any future pandemics.

### When Personal Data Are or Are Not Needed

To a large extent, how well a smart HH system is accepted depends on whether personal data are collected. We next consider what data smart HH systems can collect and what is needed for the identified actions (Table 3).

**Table 3:** Data collected by smart hand hygiene (HH) systems and whether these include personal data (from sensors that track individuals, manual input, and personal devices) or do not include personal data (from sensors in dispensers of sanitizer, soap, etc and sensors [including cameras] that track anonymized movement and location).

Data to be collected	No personal data		Personal data		
	Sensors in dispensers of sanitizer, soap, etc	Sensors (including cameras) that track anonymized movement and location within spaces	Sensors that track individuals	Manual input	Personal devices
Soap levels and soap consumption including date, time, and location of use	✓				
Correct HH technique	✓				
HH events or nonevents	✓	✓			
Date, time, duration, and location of a person’s use of a hand sanitizer	✓		✓		
Date, time, duration, and location of a person’s use of a hygiene facility	✓		✓	✓	
Contextualized use of HH facility (including what the person had been doing and where they had been)	✓	✓	✓	✓	✓

For some uses of HH data, there is no need for personal data to be collected. For resource and workflow planning and to find operating sanitizers, data about soap levels and soap consumption including date, time, and location of use can be collected from sensors in smart systems without the need to collect any personal data. Similarly, smart systems can give immediate feedback to users to improve their HH technique without collecting any personal data. It is also possible to communicate the overall standard of HH in an organization without collecting personal data. Smart system data combined with data from sensors that track movement in and out of spaces can be used to identify the extent to which all employees are practicing HH and whether they use HH facilities as they move around the building (eg, at the lift and after using the toilet). The data collected could also indicate overall compliance with organizational policy and be used to identify the overall education and training needs of the organizations. The same system could also give feedback to individuals at the point where they are using the facility.

Personal data are required for several of the uses of HH data identified in this study, particularly the uses where individuals are pinpointed such as identification of individual practices and training needs and enforcement of organizational policy. Sensors that track individuals (installed on staff ID cards, apps, or other personal devices) would be necessary to capture each person’s use of a hand sanitizer (date, time, technique, and location of use) and to send reminders. However, these data can be anonymized and aggregated to identify the uses of HH facilities by different groups (not individuals) within the organization. Good practice would be to offer a manual override that would allow employees to correct any system errors. For smart systems to fully represent a person’s HH practice, it would be necessary to allow users to manually input their use of any and all hygiene facilities such as wipes and their own sanitizer gel. To capture a person’s contextualized use of HH facility (including what the person had been doing and where they had been), the system would need to connect to other personal data such as calendars and mobile phones.

Whether personal data are needed to evaluate interventions will depend on the nature of the intervention and what needs to be evaluated. Identifying the causes of outbreaks will likely also require the collection of other personal data (eg, who is ill).

### Limitations

Through this interview study, a wide range of applications for HH data collected via smart systems has been identified. However, this is an exploratory study; further investigation is needed to determine whether office workers would use smart sanitizers for the identified purposes. Such studies could build on these findings to further investigate the implementation and adoption of smart sanitizers, with trials in offices.

### Conclusions

Smart sanitizers could, feasibly, make a contribution to the improvement of hygiene practices in offices [21], but for smart systems to be accepted, any data collected would need to be actionable [22]. This study contributes to knowledge by identifying the many potential uses for hygiene data collected from smart systems. As smart HH systems have not yet been introduced into offices, identification of constructive uses for data is important for their design and implementation.

Although smart sanitizers are widely deployed in clinical settings, health care workers recognize that HH is an important part of their professional practice [18,19]. Given that office workers consider HH to be a mostly personal matter, it seems less likely that they will want to adopt smart sanitizers. When there are disease outbreaks, office workers may consent to the sharing of personal data and the monitoring of their own and their colleagues’ HH. At other times, smart sanitizers may need to be restricted to uses that do not require any personal data collection. Should organizations wish to implement smart sanitizers in offices, it would be advisable to consult widely with the staff and to develop systems that are customizable and personalizable. It should also be noted that office workers may find it more useful to have insights from all their HH practices, but these data cannot (yet) be automatically collected from smart systems.

In contrast to health care workers, as yet, there is no widely accepted HH policy for office workers. Future studies could usefully investigate what office workers would consider to be an appropriately high standard of hygiene and how often and where hygiene should be performed. A better understanding of what would be effective and acceptable HH policies in nonclinical settings would help to clarify how smart systems can be used and hence inform their design. Importantly, it could help workplaces adopt infection prevention and control strategies that are necessary for current and future infectious disease outbreaks [1].

## Abbreviations

AHHMS: automated hand hygiene monitoring systems
HH: hand hygiene
RQ: research question

## Data Availability

This study involved human research participant data and could contain potentially identifying information. The data that support the findings of this study are available upon request from the corresponding author.
